# Systems analysis of primary Sjögren's syndrome pathogenesis in salivary glands identifies shared pathways in human and a mouse model

**DOI:** 10.1186/ar4081

**Published:** 2012-11-01

**Authors:** Steve Horvath, Abu NM Nazmul-Hossain, Rodney PE Pollard, Frans GM Kroese, Arjan Vissink, Cees GM Kallenberg, Fred KL Spijkervet, Hendrika Bootsma, Sara A Michie, Sven U Gorr, Ammon B Peck, Chaochao Cai, Hui Zhou, David TW Wong

**Affiliations:** 1 School of Dentistry, Dental Research Institute, University of California at Los Angeles, 10833 Le Conte Avenue, 73-017 CHS, Los Angeles, CA 90095-1668, USA; 2 Department of Oral and Maxillofacial Surgery, University Medical Center Groningen, University of Groningen, P.O. Box 30.001, Groningen, the Netherlands; 3 School of Medicine, Stanford University, 300 Pasteur Drive, R241 MC 5324, Stanford, CA 94305, USA; 4 School of Dentistry, University of Minnesota, 7536 Moos HST, 515 Delaware Street SE, Minneapolis, MN 55455, USA; 5 Department of Pathology, Immunology and Laboratory Medicine, School of Medicine, University of Florida College of Medicine, JHMHSC D6-33D, 1600 SW Archer Road, Gainesville, FL 32610-0275, USA

## Abstract

**Introduction:**

Primary Sjögren's syndrome (pSS) is a chronic autoimmune disease with complex etiopathogenesis. Despite extensive studies to understand the disease process utilizing human and mouse models, the intersection between these species remains elusive. To address this gap, we utilized a novel systems biology approach to identify disease-related gene modules and signaling pathways that overlap between humans and mice.

**Methods:**

Parotid gland tissues were harvested from 24 pSS and 16 non-pSS sicca patients and 25 controls. For mouse studies, salivary glands were harvested from C57BL/6.NOD-*Aec1Aec2 *mice at various times during development of pSS-like disease. RNA was analyzed with Affymetrix HG U133+2.0 arrays for human samples and with MOE430+2.0 arrays for mouse samples. The images were processed with Affymetrix software. Weighted-gene co-expression network analysis was used to identify disease-related and functional pathways.

**Results:**

Nineteen co-expression modules were identified in human parotid tissue, of which four were significantly upregulated and three were downregulated in pSS patients compared with non-pSS sicca patients and controls. Notably, one of the human disease-related modules was highly preserved in the mouse model, and was enriched with genes involved in immune and inflammatory responses. Further comparison between these two species led to the identification of genes associated with leukocyte recruitment and germinal center formation.

**Conclusion:**

Our systems biology analysis of genome-wide expression data from salivary gland tissue of pSS patients and from a pSS mouse model identified common dysregulated biological pathways and molecular targets underlying critical molecular alterations in pSS pathogenesis.

## Introduction

Sjögren's syndrome is a chronic, inflammatory autoimmune disease characterized by lymphocytic infiltration of the exocrine glands, especially the salivary and lacrimal glands, leading to destruction of their functional components. The disease may exist alone as primary Sjögren's syndrome (pSS) or in conjunction with another autoimmune disorder as secondary Sjögren's syndrome [1]. The disease affects 0.5 to 1.0% of the general population and shows a striking 9:1 female predominance [2,3]. Although dry mouth and dry eyes are the hallmark symptoms of pSS, the disease can affect almost any organ of the body and can cause substantial morbidity [4]. Symptomatic therapy currently dominates the overall management of affected individuals [5,6].

The diagnostic criteria, including the American-European Consensus Group (AECG) criteria involving either serology or histopathology in conjunction with oral and ocular dryness [7], are often inconsistently applied, and are not optimal in differentiating pSS from non-pSS sicca patients or addressing extraglandular manifestations. As a result, diagnosis of Sjögren's syndrome often lags disease onset by 6 to 10 years. Recent attempts to address these issues - the EULAR Sjögren's Syndrome Patient Reported Index, and a disease activity index to evaluate systemic complications (EULAR Sjögren's Syndrome Disease Activity Index) [8,9] - require further large-scale validation. In addition to the difficulty in disease diagnosis, the underlying pathophysiologic mechanisms of Sjögren's syndrome remain obscure.

A common model suggests interaction between genetic susceptibility and environmental factors such as viral infections for the immune cell activation and protracted inflammatory response resulting in glandular dysfunction and systemic autoimmunity [6]. In addition, recent studies in human and animal models have highlighted several components of the innate and adaptive immune systems as well as nonimmunologic factors [10-13]. However, there is a general lack of experimental and research intersections between humans and mouse models in terms of biological pathways and key molecular targets. Indeed, a wide variety of animal models of Sjögren's syndrome have been described; each capturing certain aspects of the disease [14]. The most widely used mouse model, NOD, exhibits CD4^+ ^lymphocyte infiltration, autoantibodies, xerostomia and a female-dominant phenotype. However, the mice also develop diabetes, which complicates the search for Sjögren's syndrome specific changes of gene expression. Recently, the modified NOD mouse model C57BL/6.NOD-*Aec1Aec2 *has been described. These mice develop the symptoms of Sjögren's syndrome but not diabetes [15], suggesting that they will be a highly valuable model for understanding the pathogenesis pSS.

In the present study, we applied weighted gene co-expression network analysis (WGCNA) [16] to characterize biological pathways and molecular targets associated with pSS pathogenesis in both human parotid tissue and the C57BL/6.NOD-*Aec1Aec2 *mouse model. Our systems analysis of genome-wide expression data from human (pSS) salivary gland tissue compared with salivary gland tissue from a mouse model of pSS identified common biological pathways and molecular targets that can pivotally contribute to critical molecular alterations in pSS pathogenesis.

## Materials and methods

### Human studies

The study protocol was approved by the Institutional Review Board of the University of California at Los Angeles and the University Medical Center at Groningen. All patients were recruited from the Department of Oral and Maxillofacial Surgery of the University Medical Center at Groningen, were at least 21 years old, and provided written informed consent to participate in the study. The study included 24 patients who fulfilled the 2002 AECG criteria for pSS [17], 16 non-sicca patients with subjective symptoms and objective signs of oral and ocular dryness but not fulfilling the AECG criteria for pSS, and 25 control patients with no subjective or objective evidence of oral or ocular dryness. The patient characteristics are presented in Table 1.

**Table 1:** Characteristics of primary Sjögren's syndrome, nonprimary Sjögren's syndrome sicca and control subjects

Characteristic	pSS (*n *= 24)	Non-pSS sicca (*n *= 16)	Controls (*n *= 25)
Age (years)	49.0 ± 15.8	52.8 ± 15.4	59.0 ± 12.0
Female:male	21:3	13:3	12:13
Ethnicity	24 Caucasian	16 Caucasian	25 Caucasian
Unstimulated whole saliva (ml/minute)	0.10 ± 0.14	0.12 ± 0.18	NA
Stimulated whole saliva (ml/minute)	0.31 ± 0.35	0.33 ± 0.40	NA
Schirmer's test (mm)	9.2 ± 7.6	14.8 ± 11.7	NA
Focal score	3.5 ± 1.2	0.5 ± 1.0	0
Sjögren's syndrome - A (positive:negative)	23:1	2:14	0:25
Sjögren's syndrome - B (positive:negative)	19:5	0:16	0:25

Data presented as the mean ± standard deviation. All primary Sjögren's syndrome (pSS) and non-pSS sicca patients were subjected to a complete American-European Consensus Group diagnostic work-up. NA, not assessed (in controls being head and neck cancer patients without parotid involvement, no functional tests were performed as all these patients were already scheduled for a neck dissection and functional tests were considered too much of a burden to the patients at that time).

#### Parotid gland tissue specimens

Under local anesthesia, an incisional biopsy of one parotid gland was performed as part of the diagnostic workup of the pSS and non-pSS sicca patients following AECG criteria, as described [7], and according to the procedure described by Pijpe and colleagues [18]. Tissue was harvested from the dorsal caudal lobe of the parotid gland from the control patients during surgery for oral or oropharyngeal squamous cell carcinoma.

After harvest, most of the specimen was snap-frozen and delivered to the University of California at Los Angeles on dry ice and then stored at -80°C for gene expression profiling. A minor part was fixed in 4% formalin, embedded in paraffin, sectioned, and stained with H & E for histopathologic evaluation. The evaluation of slides from pSS and non-pSS sicca patients was performed independently by two oral pathologists, who determined the focus score (≥50 lymphocytes/4 mm^2 ^glandular tissue) and other characteristics such as lymphoepithelial lesions, germinal centers, fibrosis, atrophy, and so forth. The specimens from pSS patients showed characteristic features (for example, focus score ≥1 and presence of lymphoepithelial lesions) whereas those from non-pSS sicca and control subjects did not show any such characteristics. Histopathologic evaluation of parotid tissue from controls revealed no carcinoma.

#### Gene expression profiling

The isolation of total RNA from snap-frozen parotid gland tissues, in sample sizes ranging from 10 to 30 mg, was performed using the RNeasy kit supplied with the PARIS system (Ambion, Austin, TX, USA), according to the manufacturer's protocol. Briefly, each frozen tissue specimen was homogenized in 300 μl cold cell-disruption buffer and an equal volume of 2 × denaturing solution was added. The total lysate was centrifuged for 5 minutes at 10,000 × *g *to separate out the superficial aqueous phase containing RNA and then 1.25 volumes of 100% ethanol were added. The mixture was loaded onto a spin column followed by washing twice with provided buffer. RNA was eluted at 100°C into 50 μl elution buffer. The resultant RNA was subjected to RNase-free DNase treatment followed by ethanol precipitation, dissolved into 15 μl DNase/RNase-free water, and quantified with a Nanodrop spectrophotometer (Nanodrop Technology, Wilmington, DE, USA).

The RiboAmp RNA Amplification system (Molecular Devices, Sunnyvale, CA, USA) was used to perform one round of linear amplification of parotid gland tissue mRNAs, taking 2.5 μg total RNA from each specimen as the template. The synthesized cDNA was transcribed to cRNA and then biotinylated using the GeneChip Expression 3'-Amplification reagents (Affymetrix, Santa Clara, CA, USA) for *in vitro *labeling. Then 15 μg labeled cRNA was fragmented into 50 to 200 bp fragments and assessed for quality with a 2100 Bioanalyzer (Agilent Technologies, Palo Alto, CA, USA).

The fragmented biotin-labeled cRNA from pSS, non-pSS sicca and control subjects was hybridized overnight to the Affymetrix HG U133 plus 2.0 arrays. After washing to remove the unbound transcripts, the hybridized chips were stained prior to scanning. The acquired images were processed with Affymetrix Microarray Suite software and analyzed with the R software (which is freely available at [19]).

### Mouse studies

#### Mouse model of Sjögren's syndrome-like disease

C57BL/6.NOD-*Aec1Aec2 *mice were bred and maintained under specific pathogen-free conditions within the mouse facility of the Department of Pathology at the University of Florida, Gainesville. The breeding and use of these animals for the present studies were approved by the University of Florida Institutional Animal Care and Use Committee. The animals were maintained on a 12-hour light-dark schedule and provided with food and acidified water *ad libitum*.

#### Generation of salivary gland transcriptome data

Salivary glands were freshly excised from euthanized mice (*n *= 5 per age group) at 4, 8, 12, 16, or 20 weeks of age, snap-frozen in liquid nitrogen, and stored at -80°C until all glandular samples were obtained. Each salivary gland was comprised of a submandibular, sublingual, and parotid gland minus salivary lymph nodes. Total RNA was isolated from salivary glands of each mouse using the RNeasy Mini-Kit (Qiagen, Valencia, CA, USA), in accordance with the manufacturer's protocol. Hybridizations were carried out with each of the 25 individual RNA samples using Affymetrix GeneChip Mouse Genome 430 plus 2.0 Arrays, in accordance with the manufacturer's instructions. Each GeneChip contained 45,000 probe sets that analyzed the expression level of over 39,000 transcripts and variants from over 34,000 well-characterized mouse genes.

### Statistical procedures

#### Microarray data preprocessing

The Affymetrix U133 plus 2.0 microarray data were analyzed with functions and packages of the statistical software R 2.12.0 and Bioconductor 2.7 (which can be downloaded from [19]). Expression intensity values were calculated for the 65 Human microarray CEL files (24 pSS, 16 non-pSS sicca and 25 controls) using the mas5 function of the Affy library. Potential array outliers were identified by studying their interarray correlation. After removing potential outliers in a completely unbiased fashion, all 65 samples remained for downstream analysis. After quantile normalization, the ComBat function was used to correct for batch effects [20]. To summarize multiple probe sets per gene, we used the default settings of the collapseRows R function [21]. Each of the 20,718 genes on the array was thus represented by the probe with the highest mean expression value.

Analogous preprocessing steps were applied to the 25 mouse microarray samples corresponding to five different time points (weeks 4, 8, 12, 16 and 20, each time point had five mice). We related mouse genes to human data using a table of orthologous genes.

#### Weighted gene co-expression network analysis and preservation analysis

The statistical analysis software (WGCNA R package) and R tutorials for constructing a weighted gene co-expression network can be found in the literature [16,22]. The WGCNA package first calculates all pair-wise Pearson's correlation coefficients across all samples. In a signed weighted network, the resulting Pearson's correlation matrix is transformed into an adjacency matrix [23]:

$$a(ij)=|0.5+0.5*\text{cor}(x(i),x(j)){|}^{\beta })$$

The default value of the power β = 12 facilitates a soft-thresholding approach that preserves the continuous nature of the co-expression relationships [16]. As a network dissimilarity measure we used 1 - the topological overlap measure as the input for average linkage hierarchical clustering [24]. We used the dynamic branch cutting method to define modules as branches of the hierarchical clustering tree [25]. Unassigned background genes, outside each of the modules, were denoted with the color grey. To group the 20,718 genes into modules, we used the blockwiseModule R function in the WGCNA R package.

#### Connectivity and module membership measures

Module membership (MM), also known as eigengene-based connectivity, is a measure of intramodular connectivity [26]. MM is defined as:

MM(i)=cor(x(i),ME)

where *x(i*) is the expression profile of *i*th gene and ME is the eigengene (first principal component) of the given module. We used the MM measure to select module genes for a gene ontology (GO) enrichment analysis. The MM measures of the human modules are reported in Additional file 1.

#### Functional enrichment analysis

The Database for Annotation, Visualization, and Integrated Discovery (available at [27]) and the Ingenuity Pathways Analysis software (Ingenuity Systems available at [28]) were used to determine whether sets of genes (for example, preserved intramodular hub genes) were significantly enriched with known GOs. The Ingenuity software only reports uncorrected *P *values. The functional enrichment results are reported in Additional file 2.

#### Module preservation analysis

To evaluate which human modules could also be found in the mouse data, we used module preservation statistics implemented in the modulePreservation R function [17]. For each module in the reference data (for example, human data), a permutation test leads to the Zsummary statistic in the test data (here the mouse dataset). Zsummary >10 indicates strong evidence of preservation while Zsummary <2 indicates no preservation according to the permutation test.

#### Standard differential expression analysis

To find differentially expressed genes between pairs of groupings (involving pSS, sicca, controls), we used two R functions in the WGCNA package (standardScreeningBinaryTrait and standardScreeningNumericTrait) that report *P *values, false discovery rates (*q *values), fold changes and other widely used statistics for selecting differential expressed genes. Additional file 3 reports the differential expression results in humans for studying contrasting pSS versus controls, sicca patients versus controls, and pSS versus sicca patients, and for relating expression data to gender in control subjects. Further, Additional file 3 reports the correlation of each gene with an ordinal measure of diagnosis (0 = controls, 1 = sicca, 2 = pSS), which allows the reader to identify genes that positively or negatively increase with the disease progression in humans. We also correlated each mouse gene with time (weeks) to find genes that are increasing or decreasing as time progresses. These results (*P *values, correlations) are reported in Additional file 4. These results were also used to create Table 2.

**Table 2:** Genes with concordant progression patterns in human and mouse disease

Progression	GeneSymbol	corHuman	p.Human	corMouse	p.Mouse	MM.magenta
+	ADA	0.57	5.5 × 10^-7^	0.62	9.2 × 10^-4^	0.70
+	AIF1	0.51	1.6 × 10^-5^	0.57	3.1 × 10^-3^	0.67
+	C1QB	0.54	3.2 × 10^-6^	0.65	4.6 × 10^-4^	0.71
+	CASP3	0.59	1.8 × 10^-7^	0.52	8.2 × 10^-3^	0.59
+	CYBB	0.63	1.6 × 10^-8^	0.56	3.4 × 10^-3^	0.82
+	ENTPD1	0.51	1.2 × 10^-5^	0.62	8.5 × 10^-4^	0.66
+	GZMA	0.63	1.7 × 10^-8^	0.70	1.0 × 10^-4^	0.72
+	GZMK	0.61	5.8 × 10^-8^	0.55	4.1 × 10^-3^	0.63
+	HLA-DQB1	0.53	6.0 × 10^-6^	0.65	5.0 × 10^-4^	0.48
+	HLA-DRB1	0.53	5.9 × 10^-6^	0.74	2.6 × 10^-5^	0.89
+	IFIT1	0.59	2.6 × 10^-7^	0.59	1.9 × 10^-3^	0.43
+	ITGAX	0.51	1.2 × 10^-5^	0.63	6.8 × 10^-4^	0.68
+	LY86	0.58	4.4 × 10^-7^	0.53	6.4 × 10^-3^	0.78
+	PLEKHA2	0.63	1.5 × 10^-8^	0.52	7.1 × 10^-3^	0.87
+	STAT1	0.67	7.2 × 10^-10^	0.64	5.2 × 10^-4^	0.60
+	TLR7	0.63	1.6 × 10^-8^	0.63	6.6 × 10^-4^	0.61
-	ALDH3A2	-0.50	1.8 × 10^-5^	-0.63	8.0 × 10^-4^	-0.64
-	ANGPTL4	-0.50	1.9 × 10^-5^	-0.58	2.6 × 10^-3^	-0.40
-	ATP1B1	-0.56	1.0 × 10^-6^	-0.58	2.5 × 10^-3^	-0.62
-	CPT1A	-0.54	4.0 × 10^-6^	-0.76	8.7 × 10^-6^	-0.55
-	FAF1	-0.54	4.1 × 10^-6^	-0.55	4.5 × 10^-3^	-0.58
-	NEO1	-0.52	9.6 × 10^-6^	-0.53	6.9 × 10^-3^	-0.47
-	PALMD	-0.50	2.2 × 10^-5^	-0.64	6.1 × 10^-4^	-0.65
-	PDHA1	-0.58	3.9 × 10^-7^	-0.63	8.0 × 10^-4^	-0.71
-	PPFIBP2	-0.56	1.2 × 10^-6^	-0.57	3.2 × 10^-3^	-0.48

corHuman denotes the correlation of the gene with human progression status (defined as 0 for controls, 1 for sicca, and 2 for primary Sjögren's syndrome). corMouse denotes the correlation of the gene with mouse progression status (weeks). p.Human and p.Mouse report the correlation test *P *values in human and mouse data, respectively. MM.magenta reports the module membership value of the gene.

## Results

### Identification of gene co-expression modules in parotid glands of pSS patients

**Figure 1:**
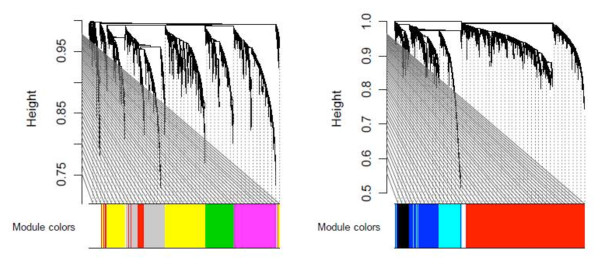
**Hierarchical cluster trees for detecting modules based on weighted gene co-expression network analysis**. All array samples (primary Sjögren's syndrome patients, sicca, and controls) were to find co-expression modules. Modules correspond to branches of the trees and are assigned color categories as indicated by the color band underneath each tree (see Materials and methods). The two panels correspond to two cluster trees resulting from the blockwiseModules WGCNA R function [21], which was used to circumvent computational challenges. Each cluster tree thus corresponds to a block of genes.

A signed weighted gene co-expression network was constructed based on the 65 human parotid gland tissues (24 pSS, 16 non-pSS sicca and 25 controls). The WGCNA method clustered the 20,718 human genes into 19 distinct gene co-expression modules. Since the module detection is unbiased and does not make use of GO information, each of the modules was initially labeled with a unique color as an identifier (Figure 1). To define a representative module expression profile (referred to as the module eigengene), we summarized the (standardized) gene expression profiles of the module eigengene (= first principal component). The module eigengene can be considered a weighted average of the module gene expression profiles. To identify disease-related modules, we correlate each module eigengene with disease status.

**Figure 2:**
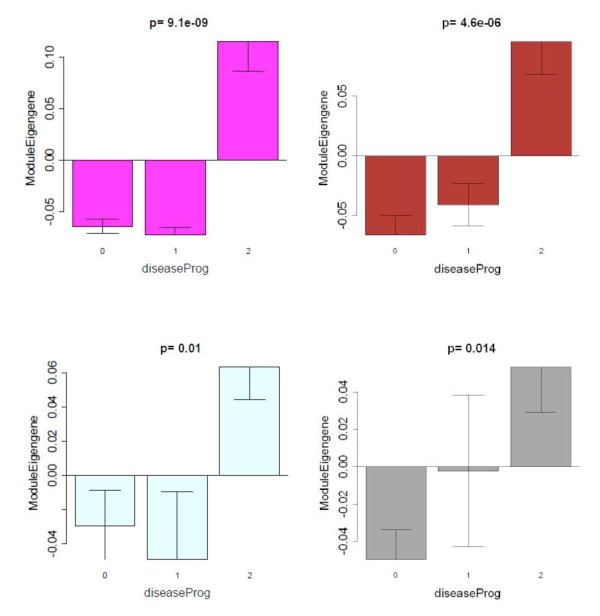
**Identification of primary Sjögren's syndrome disease-related gene modules revealed by weighted gene co-expression network analysis**. Barplots showing the mean value of the module eigengene (*y *axis) versus human disease progression status (*x *axis) where 0 denotes healthy controls, 1 denotes nonprimary Sjögren's syndrome (non-pSS) sicca patients, and 2 denotes pSS patients. Kruskal-Wallis test *P *values above the plots show that the module eigengenes of all four modules - Magenta (576 genes), Brown (2,502 genes), Grey60 (216 genes), and Light-Cyan (349 genes) - are significantly overexpressed in pSS patients compared with both non-pSS sicca patients and controls.

**Figure 3:**
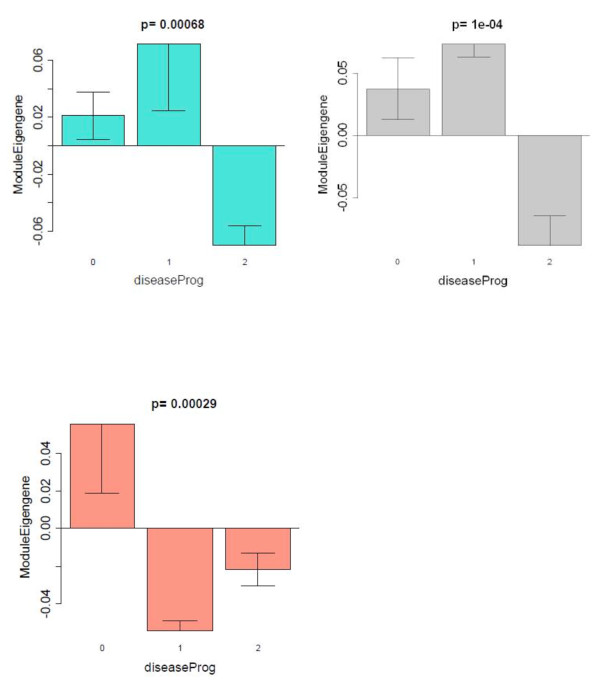
**Primary Sjögren's syndrome disease-related co-expression modules that are downregulated in primary Sjögren's syndrome patients**. Barplots analogous to those described in Figure 2. The module eigengenes of two modules - Turquoise (3,981 genes) and Grey (3,011 genes) - are significantly underexpressed in primary Sjögren's syndrome (pSS) patients compared with both non-pSS sicca patients and controls. Note that the Salmon module (446 genes) differs from the other two modules with respect to the non-pSS sicca patients, where the module eigengene is underexpressed compared with controls.

Strikingly, seven out of 19 modules showed significant differential expression between pSS and control samples, which reflects the fact that thousands of genes are differentially expressed between the two groups. In particular, we found a highly significant positive correlation between disease and the Magenta (comprised of 576 genes) module eigengene (*r *= 0.67, *P *<2 × 10^-7^), the Brown (2,502 genes) module eigengene (*r *= 0.6, *P *<6 × 10^-6^), the Light-Cyan (349 genes) module eigengene (*r *= 0.42, *P *<0.002), and the Grey60 (based on 216 genes) module eigengene (*r *= 0.47, *P *<8 × 10^-4^). Since a positive correlation indicates that the corresponding module genes are overexpressed in the diseased individuals, the Magenta, Brown, Light-Cyan and Grey60 modules are comprised of genes overexpressed in pSS samples compared with both non-pSS sicca and controls (Figure 2). On the contrary, we found a highly significant negative correlation between disease status and the Turquoise (based on 3,981 genes) module eigengene (*r *= -0.52, *P *<10^-4^), the Grey (based on 3,011 genes) module eigengene (*r *= -0.41, *P *< 0.003), and the Salmon (based on 446 genes) module eigengene (*r *= -0.28, *P *<0.005). The Turquoise, Grey and Salmon modules are thus comprised of genes that are underexpressed in pSS samples compared with both non-pSS sicca and controls (Figure 3). All of these *P *values remain highly significant even after carrying out the most stringent multiple comparison adjustment (Bonferroni correction) for the number of modules. This co-expression module-based analysis has a major advantage over a standard differential gene expression analyses since it only relates 19 modules to pSS disease status, alleviating the multiple comparison problem inherent in the raw data.

For each gene, Additional file 1 reports MM measures (also known as eigengene-based connectivity) between genes and modules.

### Preservation of human Magenta module in a mouse Sjögren's syndrome model

We applied the modulePreservation R function to assess whether modules defined by the human data were preserved in C57BL/6.NOD-*Aec1Aec2 *mice, Sjögren's syndrome-like disease model datasets. We selected the C57BL/6.NOD-*Aec1Aec2 *mice for the comparative gene expression analysis because it is a spontaneous disease model, probably the best-defined mouse model to date with respect to its disease profile, and has a comparative control with the same genetic background that mimics virtually all molecular and clinical aspects defined in humans. Importantly for this study, extensive transcriptomic profiling data of salivary glands defining development of Sjögren's syndrome are available.

The module preservation statistics allow one to quantify which aspects of within-module topology are preserved between a reference network (human parotid glands) and a test network (mouse salivary glands). We used two composite preservation statistics (Zsummary and medianRank) to assess overall module preservation. We evaluated the preservation of the human modules in the entire mouse dataset (that is, all weeks together). Strikingly, both statistics revealed that the human Magenta module is the most highly preserved module in the mouse data (for example, Zsummary = 11, *P *<10^-18^). Further, Additional file 5 demonstrates that the Magenta module eigengene in the mouse data shows increasing expression across times; that is, the Magenta genes are significantly (*P *= 0.034) overexpressed in weeks 16 and 20. These results indicate that the mouse model is appropriate when it comes to an aspect of the human disease embodied in the Magenta module.

### Gene ontology and pathway enrichment analysis

To study the ontology of genes in the seven human pSS related co-expression modules, we used the Database for Annotation, Visualization and Integrated Discovery and the Ingenuity Pathway Analysis software. The enrichment results are reported in Additional file 2. Here, we highlight results for the two most significant modules: Magenta and Turquoise.

For the Magenta module, the top-three over-represented subcategories within 'Gene Ontology - Biological Process' were immune response (115 genes out of 423 Magenta module genes in both human and mouse datasets, *P *= 1.58 × 10^-52^, Bonferroni-corrected *P *= 3.39 × 10^-49^), defense response (*P *= 2.69 × 10^-27^, Bonferroni-corrected *P *= 5.76 × 10^-24^) and inflammatory response (46 genes in both human and mouse dataset, *P *= 1.96 × 10^-17^, Bonferroni-corrected *P *= 4.20 × 10^-14^). Additional enriched categories for the Magenta module include antigen processing and presentation (*P *= 2.95 × 10^-17^), positive regulation of response to stimulus (*P *= 4.30 × 10^-17^), cell adhesion molecules (*P *= 1.34 × 10^-13^), humoral immune response (*P *= 2.38 × 10^-13^), response to wounding (*P *= 5.68 × 10^-13^), lymphocyte activation (*P *= 2.93 × 10^-11^), and cell activation (*P *= 9.03 × 10^-11^).

**Figure 4:**
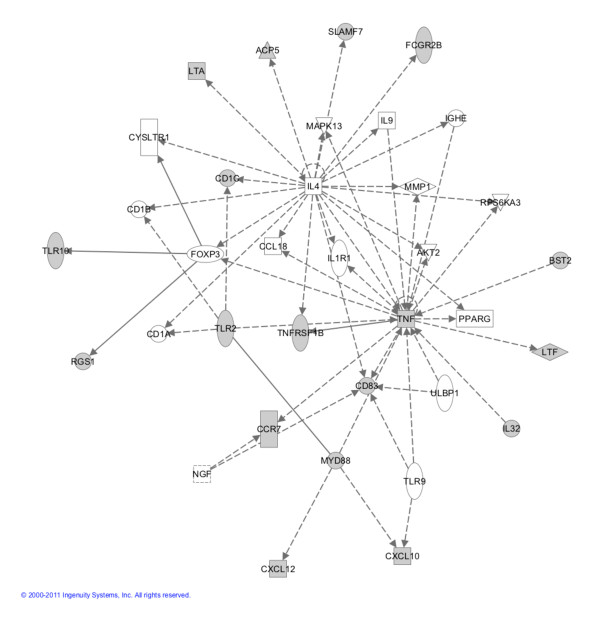
**Ingenuity Pathway Analysis software network plot**. Genes shaded grey were part of the magenta module and are significantly altered in the parotid gland of primary Sjögren's syndrome patients. Test statistics and *P *values of individual genes can be found in Additional file 3 (humans) and Additional file 4 (mouse).

Strikingly, four of the Magenta genes are implicated the IL-4 signaling pathway (*P *= 0.025). Figure 4 shows an Ingenuity Pathway Analysis network plot where genes colored in grey are part of the Magenta module. Genes identified include those that encode for Toll-like receptors and their signal transduction molecule MyD88, molecules that present antigen to natural killer cells (for example, members of CD1), molecules involved in immune cell recruitment and adherence (for example, CCL21, CXCL10, CXCL12, CCR7 and SLAM7), and molecules responsible for formation of germinal centers in secondary lymphocyte organs,for example, lymphotoxin alpha (LTA). CXCL10 (or IP10), CXCL12 (or stromal cell-derived factor-1) and SLAM7 are of particularly interest as they indicate recruitment of specific leukocyte subsets - in particular, macrophages, dendritic cells, T and B lymphocytes, and natural killer cells. We have independently validated these six genes using quantitative PCR (Additional file 6).

For the Turquoise module where the genes were underexpressed in pSS, the top-three over-represented subcategories within 'Gene Ontology - Biological Process' were oxidation reduction (*P *= 7.89 × 10^-11^, Bonferroni-corrected *P *= 3.74 × 10^-7^), generation of precursor metabolites and energy (*P *= 4.72 × 10^-9^, Bonferroni-corrected p = 2.24 × 10^-5^) and cofactor metabolic process (*P *= 1.45 × 10^-6^, Bonferroni-corrected *P *= 0.0069). The category 'KEGG pathway' yielded the following top-three categories: oxidative phosphorylation (*P *= 9.12 × 10^-7^, Bonferroni-corrected *P *= 1.77 × 10^-4^), Alzheimer's disease (*P *= 5.66 × 10^-6^, Bonferroni-corrected *P *= 0.0011) and Huntington's disease (*P *= 3.69 × 10^-5^, Bonferroni-corrected *P *= 0.0071).

Additional files 3 and 4 report the results from a standard differential expression analysis. These look-up tables allow the user to identify genes strongly related to disease status in humans and mice, respectively. For example, Table 2 reports genes with concordant progression patterns in human and mouse disease. Positively related genes were selected by requiring a correlation >0.5 with human progression status (defined as 0 for controls, 1 for sicca, and 2 for pSS) and a correlation >0.5 with mouse progression status (weeks). Similarly, we selected the negatively correlated genes using a correlation threshold of -0.5. The corresponding *P *values are reported in Table 2. The MM column (MM.magenta) shows that most of these genes have concordant pattern with the Magenta module eigengene, illustrating again that this module contains genes that are involved in both human and mouse disease pathology.

## Discussion

We have utilized a systems approach to identify the molecular and cellular events underlying the pathogenic process in parotid glands of pSS patients, and compared the altered expressed genes and key signaling pathways with those in the salivary glands of mice that mimic the features of pSS. Using a robust statistical and bioinformatics tool, WGCNA, coupled with GO and Ingenuity Pathways Analysis software, we have clustered hundreds of co-expressed genes into 19 gene modules. Since the module definition does not make use of GO information, the modules are initially named by a color.

Our analyses highlighted seven co-expressed modules that were enriched with genes that could discriminate pSS patients from unaffected individuals using parotid gland tissue, a prime target in Sjögren's syndrome pathogenesis. Interestingly, four of the seven modules - namely, Magenta, Brown, Light-Cyan and Grey60 - were positively correlated with the disease status, suggesting that the genes contained in these modules are overexpressed in pSS. On the other hand, Turquoise, Gray and Salmon modules were negatively correlated with the disease status, suggesting that the genes in these modules are underexpressed in pSS.

We acknowledge that clinical samples are not gender-matched between the Sjögren's subjects and the non-Sjögren's subjects, which may be a potential source of bias. We have found that gender has a negligible effect on gene expression levels in the controls. None of the genes were gender related at a false discovery rate threshold of 0.05. Of the 5,461 genes that were differentially expressed between pSS cases and controls at a false discovery threshold of 0.05, only 140 genes showed differential expression (*P *<0.05) between males and females of the control samples. Further, we find that the vast majority of module genes are on autosomes and that there is no evidence that gender affects their expression level in the control group.

When comparing human and mouse data, we found that the Magenta module was the most highly conserved module between these two species. Not surprisingly, this module was enriched with genes involved in immunity and inflammation - the two cardinal events in pSS pathogenesis. To our knowledge, we are the first to map out important intersects between human and mouse pertaining to key molecules and associated pathways in pSS. The knowledge gained from this work will enhance future target-based therapies for this devastating disorder. Our novel co-expression module-based comparison of human and mouse models can be used to judge whether a given mouse model mirrors the human disease at the transcriptional level. Future studies could explore whether other mouse models allow for the preservation of additional human disease-related modules.

Characterizing the molecular and cellular events during the progression of pSS from a healthy or even a non-pSS state remains an important challenge. Because of the lack of specific molecular markers, it is difficult to determine which healthy subject or sicca patient will progress to pSS. The gain in knowledge of these events can suggest which non-pSS individuals are at risk for developing pSS. In addition, the knowledge base could be used to intervene in the progression of disease by target-based therapies. Surprisingly, such specific regimens are not in place at present, still relying on a few trials with B-cell-targeted and TNF-directed therapies evolved from testing on other autoimmune diseases [6,10,29-32]. Our systems-level analyses of high-throughput gene expression data can demystify the critical molecular alterations in pSS pathogenesis.

WGCNA is a tool of systems biology analysis and has proven to be instrumental in identifying biological pathways and key gene constituents in a number of diseases [16,22,26,33,34]. Our module-based analysis not only alleviated the multiple testing problems inherent in microarray data analysis, but also identified biologically plausible, pSS-related modules that are highly significantly enriched with relevant GO categories.

The GO analyses revealed striking correlations, both positive and negative, between gene modules and disease status. The most positively correlated Magenta module was significantly enriched with genes involved in antigen processing and presentation, and immune and inflammatory responses. Antigen processing and presentation are two important events in innate immunity in relation to pSS, and our findings support these two phenomena [31]. Interestingly, a few Magenta genes were found to be part of the IL-4 signaling pathway, which interconnected with the TNF pathway that has been implicated in pSS disease progression. The negatively correlated Turquoise module is enriched with genes underexpressed in pSS, including those involved in oxidation reduction, generation of precursor metabolites and energy, and cofactor metabolic process. pSS patients are known to have reduced energy levels, while chronic fatigue syndrome is a common extraglandular manifestation [35]. Our data seem in line with this clinical presentation of the disease.

A unique feature of the present study is the direct comparison of the human gene expression data with that of the Sjögren's syndrome-susceptible C57BL/6.NOD-*Aec1Aec2 *mouse model. Since only the human disease-related Magenta module was highly significantly preserved in the mouse data, we focused on the Magenta module to identify disease-relevant pathways and target genes common to both species. A majority of the identified genes were part of the immune response, whereas a minor fraction was part of the inflammatory response. Further comparison between overexpressed genes in human and increasingly expressed genes during the disease time course in mouse led to the identification of CD1, CCR7, CXCL10 (or IP10) CXCL12 (or stromal cell-derived factor-1), SLAM7 and lymphotoxin alpha (LTA). These genes are of particular interest as they indicate recruitment of specific leukocyte subsets - in particular, macrophages, dendritic cells, T and B lymphocytes, and natural killer cells - or are involved in germinal center formation [36]. These observations provide a compelling concept that our systems approach can identify targets and pathways overlapped in human and mouse, supporting the concept that these overlapping genes and their associated pathways are critical for pSS disease development and manifestations of clinical disease. We have independently validated these six genes using quantitative PCR (Additional file 6).

## Conclusion

Weighted gene co-expression network analysis identified a pSS-related co-expression module that relates to pSS disease not only in human parotid gland but also in mouse salivary gland gene expression data. The key genes that are part of the human-mouse intersection network are useful for elucidating critical pathways and molecular alterations dysregulated in pSS pathogenesis, for highlighting genes that could be a major focus of rodent-based validation studies, and ultimately for developing therapeutic targets in this debilitating disease. This systems biologic study also illustrates how comparative WGCNA analysis (based on module preservation statistics) can be used to assess the suitability of a rodent model with respect to human disease-related transcriptional changes.

## Abbreviations

AECG: American-European Consensus Group; bp: base pair; EULAR: European League against Rheumatism; GO: gene ontology; H & E: hematoxylin and eosin; IL: interleukin; MM: module membership; PCR: polymerase chain reaction; pSS: primary Sjögren's syndrome; TNF: tumor necrosis factor; WGCNA: weighted gene co-expression network analysis.

## Competing interests

DTWW is the co-founder of RNAmeTRIX Inc., a molecular diagnostic company. The remaining authors declare that they have no competing interests.

## Authors' contributions

SH, ANMN-H, AV, SAM, SUG, ABP and DTWW participated in the design of the study. ANMN-H, RPEP, FGMK, AV, CGMK, FKLS, HB and ABP were involved with the data acquisition. SH, CC, AV, ABP and HZ performed the statistical analyses. SH, ANMN-H, RPEP, FGMK, AV, CGMK, FKLS, SAM, SUG, ABP and DTWW drafted the manuscript. All authors read and approved the final manuscript.

## Supplementary Material

Additional file 1 **a table presenting MM membership for human data**. For each gene, the table reports the MM measure, which is also known as eigengene-based connectivity. Each module gives rise to its own MM measure; for example, MM denotes the measure for the magenta module. Columns whose name starts MM report the Pearson correlation coefficient between the gene expression value and the respective module eigengene. For each MM measure (a correlation coefficient), one can also report a corresponding correlation test *P *value based on the Student *t *test (see columns whose name starts p.MM). For example, p.MM.magenta reports a two-sided correlation test *P value *based on the Student *t *distribution. Column B reports the original module assignment based on the hierarchical cluster tree but the module membership measures were used to select genes for the functional enrichment analysis. Click here for file

Additional file 2 **a table presenting GO terms for genes in human pSS-associated co-expression modules**. The table reports GO categories, uncorrected test *P *values, and corresponding *P *values that correct for multiple comparisons using the following methods: Bonferroni, Benjamini, and the false discovery rate. It also reports the number of population hits and related count data used to calculate the hypergeometric test *P *value. Genes column reports the Affymetrix probes that were hits for the corresponding GO term. The magenta module (highlighted in yellow) is of particular interest since it was preserved in the mouse model. Click here for file

Additional file 3 **a table presenting standard differential expression analysis in human data. For each gene (transcript), the results of several two-group comparison tests (carried out with the WGCNA function standardScreeningBinaryTrait) are reported**. The first group comparison test contrasts females versus males in control subjects only (columns B through L). The second group comparison test compares controls versus pSS (columns M through W). The third group comparison test compares controls versus sicca (columns × through AH). The fourth group comparison test compares pSS versus sicca (columns AI through AS). Further, columns AT through AZ report the results of a correlation test where each gene was correlated with an ordinal variable that encodes disease status (0 for controls, 1 for sicca, and 2 for pSS). Any column whose name is preceded by corPearson reports the Pearson correlation coefficients where the binary grouping variable (for example, female vs. males) was coded as a binary numeric variable (1 for the first group, 0 for the second group). The meaning of the first and second groups can be learned from column t.Student, which reports the Student *t*-test statistic. For example, t.Student.F.vs.MGenderInControls shows that the first group corresponds to females and the second group to males (among the control samples). Similarly, t.Student.Control.vs.pSS and t.Student.Control.vs.Sicca show that the first group is comprised of controls. t.Student.pSS.vs.Sicca shows that the first group is comprised of pSS patients. Column meanFirstGroup reports the mean expression value in the first group. FoldChange column reports a signed fold-change value defined by the ratio meanFirstGroup/meanSecondGroup if meanFirstGroup >meanSecondGroup. But if meanFirstGroup <meanSecondGroup, the fold change is defined as minus meanSecondGroup/meanFirstGroup. SE.FirstGroup reports the standard error in the first group. AreaUnderROC reports the area under the receiver operating characteristic curve. pvalueStudent reports the Student *t *test *P *value that corresponds to the Student t-test statistic. q.Student reports the corresponding *q *value (local false discovery rate) calculated with the qvalue R package. nPresentSamples reports the number of nonmissing observations that were available. Click here for file

Additional file 4 **a table presenting correlations of mouse expression data with time**. This comma-delimited file reports the results from a correlation test where each mouse gene (transcript) is correlated with time (measured in weeks). Column corTime reports the correlation coefficient, column ZCorTime reports the corresponding Student *t*-test statistic, and column pValueStudentCorTime reports the corresponding two-sided Student *t *test *P *value. Column AreaUnderROCCorTime reports the area under the receiver operating characteristic curve calculated with the function rcorr.cens in the Hmisc R package. Click here for file

Additional file 5 **a figure showing Magenta module expression in the mouse data**. For each week (*x *axis) the height of the bar shows the mean of the magenta module eigengene value (±1 standard error). *P *value calculated with the Kruskal-Wallis test, which is a nonparametric group comparison test. While the magenta module was defined based on the human data, this plot shows how the corresponding module eigengene relates to time course in the mouse data. To define the magenta module eigengene in the mouse data, human genes were mapped to orthologous mouse genes. Click here for file

Additional file 6 **a figure showing the quantitative real-time PCR validation in the mouse data**. The results of the quantitative RT-PCR validation analysis involving a select group of genes (CD1D1 ortholog of CD1D, CCR7, CXCL10, CXCL12, SLAMF9, LTA) are reported. Barplots in the upper and lower panels correspond to the expression values measured by microarrays and RT-PCR, respectively. The bars are colored according to time (weeks). To verify the selected gene expressions (*n *= 7), aliquots of salivary gland RNA originally used for the microarray data were pooled. Each cDNA preparation was quantified by spectrophotometry and PCR performed. Quantifications were determined by ImageJ. Relative gene expression values yielded by the PCR arrays are compared directly with data yielded by the Affymetrix 3' Expression Array GeneChip Mouse Genome 430 2.0 arrays. Pooling RNA from each time point prior to cDNA preparation is thought to be the underlying reason for higher transcript detection in a couple of RT-PCR reactions (for example, in Cxcl12 samples). Click here for file
