# Supplementary figures and images for: Systems analysis of primary Sjögren's syndrome pathogenesis in salivary glands identifies shared pathways in human and a mouse model
Source: Arthritis Res Ther. 2012 Nov 1;14(6):R238. doi: 10.1186/ar4081 (PMC3674589; doi:10.1186/ar4081)

# Magenta eigengene expression in mice $p=0.034$

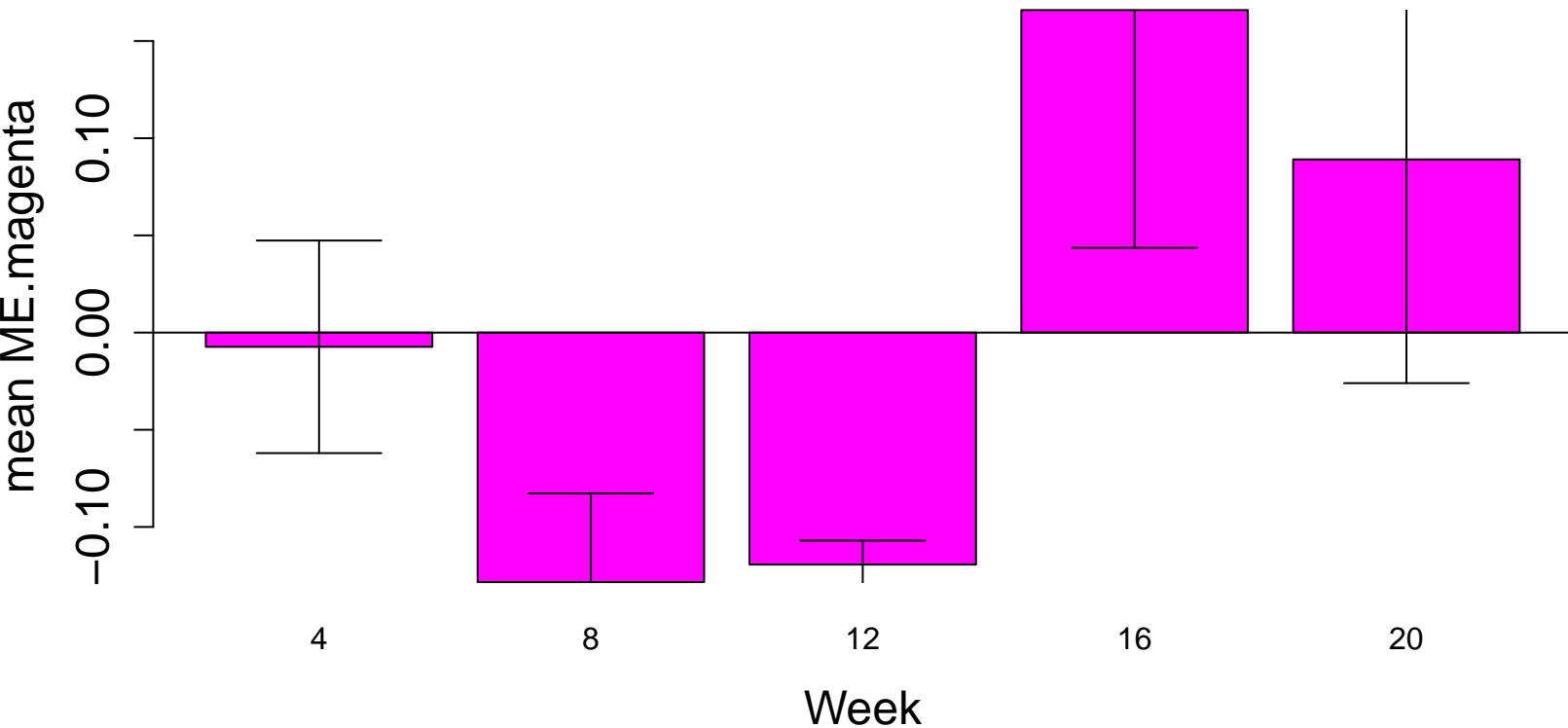

Supplement: Additional file 5 — a figure showing Magenta module expression in the mouse data. For each week (x axis) the height of the bar shows the mean of the magenta module eigengene value (±1 standard error). P value calculated with the Kruskal-Wallis test, which is a nonparametric group comparison test. While the magenta module was defined based on the human data, this plot shows how the corresponding module eigengene relates to time course in the mouse data. To define the magenta module eigengene in the mouse data, human genes were mapped to orthologous mouse genes. [file ar4081-S5.PDF]
