# Supplementary material for: Systems analysis of primary Sjögren's syndrome pathogenesis in salivary glands identifies shared pathways in human and a mouse model
Source: Arthritis Res Ther. 2012 Nov 1;14(6):R238. doi: 10.1186/ar4081 (PMC3674589; doi:10.1186/ar4081)
Supplement: Additional file 6 — a figure showing the quantitative real-time PCR validation in the mouse data. The results of the quantitative RT-PCR validation analysis involving a select group of genes (CD1D1 ortholog of CD1D, CCR7, CXCL10, CXCL12, SLAMF9, LTA) are reported. Barplots in the upper and lower panels correspond to the expression values measured by microarrays and RT-PCR, respectively. The bars are colored according to time (weeks). To verify the selected gene expressions (n = 7), aliquots of salivary gland RNA originally used for the microarray data were pooled. Each cDNA preparation was quantified by spectrophotometry and PCR performed. Quantifications were determined by ImageJ. Relative gene expression values yielded by the PCR arrays are compared directly with data yielded by the Affymetrix 3' Expression Array GeneChip Mouse Genome 430 2.0 arrays. Pooling RNA from each time point prior to cDNA preparation is thought to be the underlying reason for higher transcript detection in a couple of RT-PCR reactions (for example, in Cxcl12 samples). [file ar4081-S6.DOCX]

### Additional File 6:


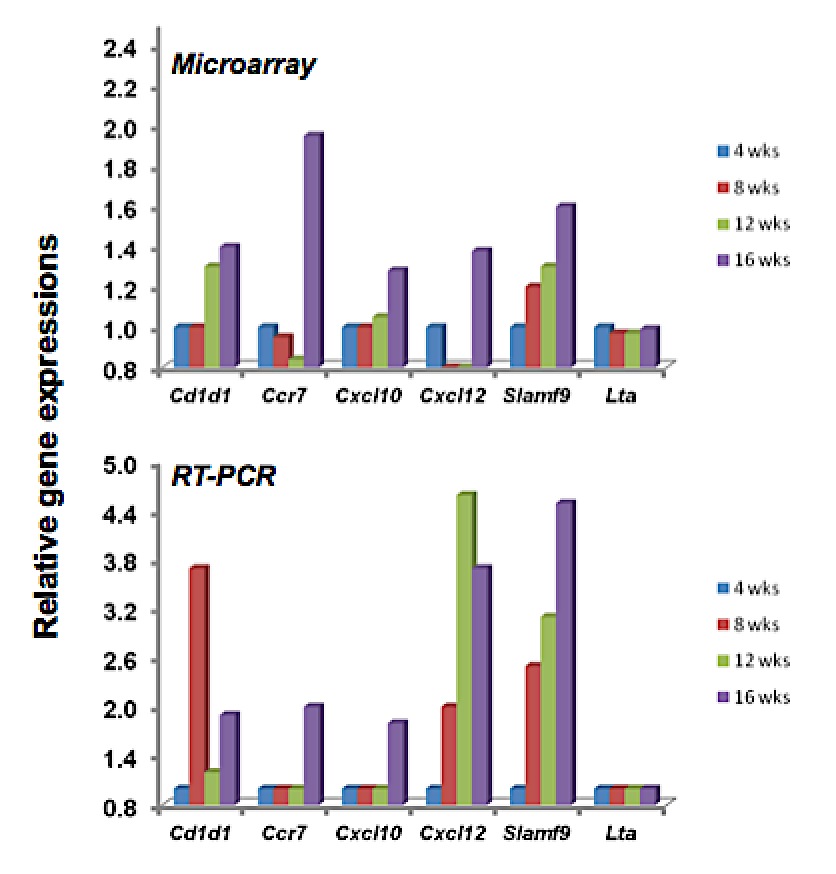


### Verification of selected gene expression

Previous verifications of microarray gene expression data using randomly selected genes have been carried out using semi-quantitative polymerase chain reaction (RT-PCR) analysis [[34](#_ENREF_34), [35](#_ENREF_35)], quantitative real-time PCR (rt-PCR) analysis [[35](#_ENREF_35), [36](#_ENREF_36)], and comparative microarray analyses using SABioscience arrays [[37](#_ENREF_37)]. These analyses have provided excellent correlations. To verify a set a selected gene expressions (n=7), aliquots of salivary gland RNA originally used for the microarray data were pooled, then prepared as described elsewhere [[36](#_ENREF_36)]. Each cDNA preparation was quantified by spectrophotometry and PCR performed. Quantifications were determined by ImageJ. Relative gene expression values yielded by the PCR arrays are compared directly with data yielded by the Affymetrix 3’ Expression Array GeneChip Mouse Genome 430 2.0 arrays. Pooling RNA from each time point prior to cDNA preparation is thought to be the underlying reason for higher transcript detection in a couple of RT-PCR reactions, e.g., in Cxcl12 samples.
